# Supplementary material for: On Benchmarking Human-like Intelligence in Machines
Source: Open Mind (Camb). 2026 Aug 21;10:1394–412. doi: 10.1162/OPMI.a.385 (PMC13618755; doi:10.1162/OPMI.a.385)
Supplement: Supplementary file 1 [file opmi-10-1394-s001.pdf]

Supplementary Materials:
On Benchmarking Human-like Intelligence in Machines

A Experiment Design

A.1 Dataset sources

The BigBench dataset consists of 204 tasks. Among the tasks we used in the evaluation study, the Social Support task is adapted from a dataset published by Wang and Jurgens (2018). The Social IQA task is taken from Sap et al. (2019). Other tasks are constructed from various online sources. We refer to BigBench (Srivastava et al., 2022) for detailed descriptions. All datasets have MIT license or Apache license. The use of these datasets in our study is permitted under these licenses.

A.2 Converting multiple choices to soft labels

All benchmarks used in our experiment provide one single answer key with 2–4 answer options for each stimulus. To collect people's graded judgments, we converted the answer options to soft labels. For binary Yes/No questions (e.g. whether a statement is supportive), we use a single scale (e.g. 1 = extremely not supportive, 100 = extremely supportive). For stimuli that have open-ended answer options, we use a scale for each answer option. For example, consider the following stimulus:

```
After rushing to make it to the gate, Robin missed his flight,
so Cameron picked him up from the airport.
What will happen to Robin?

A. Be in a car
B. Pick up their friend
C. Be on a plane
```

For each of the three answer options, the participants answer by dragging a scale (1 = definitely disagree, 100 = definitely agree). An example of the slider can be found in Figure 1.

A.3 Evaluation metrics

We compute human average accuracy per task as the percentage of trials where a participant's response agreed with the benchmark's ground-truth label. For multi-option tasks (e.g., Social IQA, Simple ethical questions, BigToM, Ambiguous Story Task), where participants rate each answer option on a separate slider, agreement is determined by whether the participant's highest-rated option (argmax) matches the ground-truth label. For binary or bipolar tasks (e.g., Dark humor detection, Irony identification, Moral permissibility), where participants provide a single slider response, agreement is determined by whether the slider value falls on the same side of the 50-point midpoint as the ground-truth label. For Social support, which uses a tripolar scale

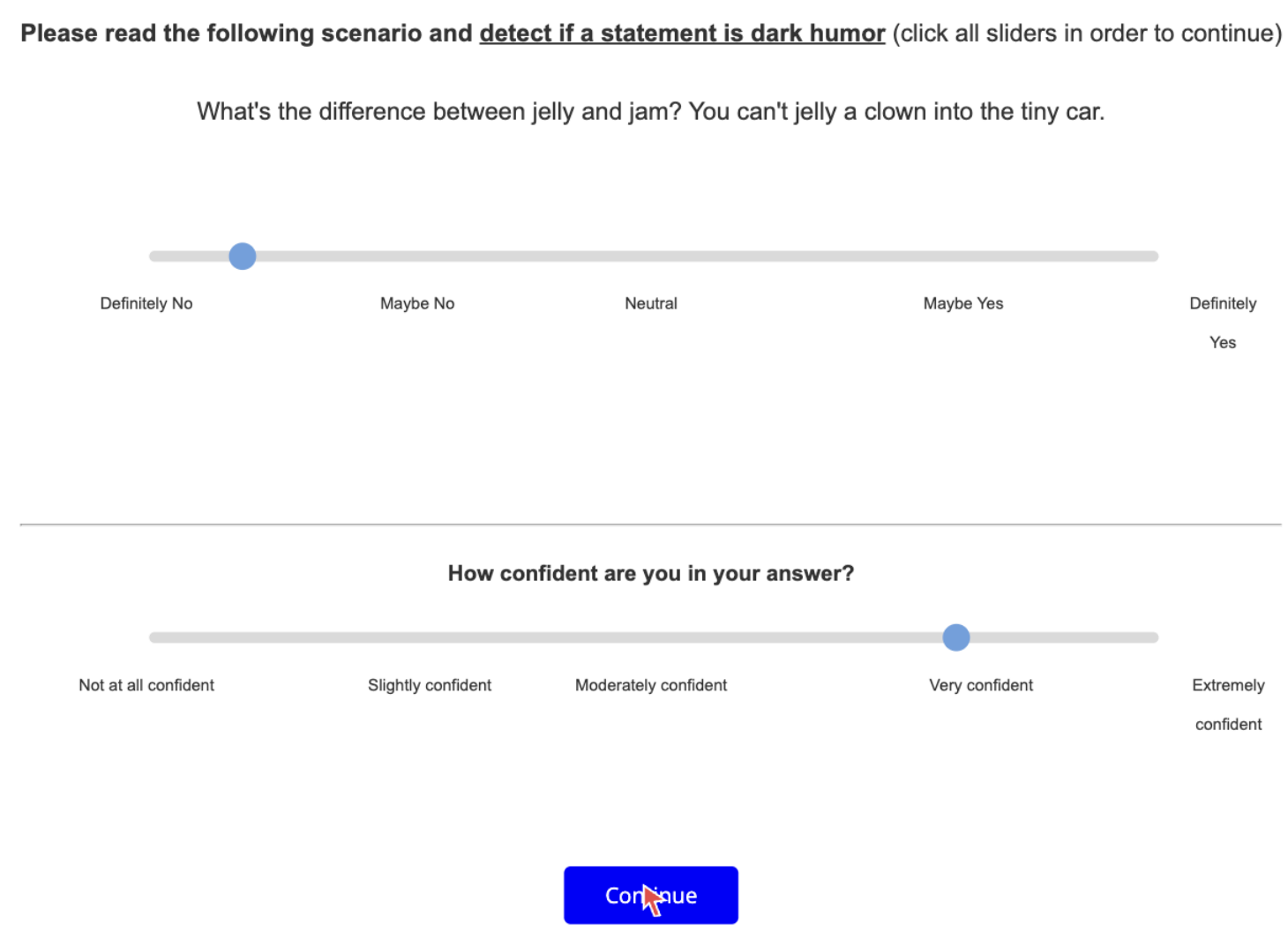

Figure 1: A screenshot of the experiment interface. Participants provide a graded judgment on the task using a slider (top), followed by a confidence rating on a separate slider (bottom).

(unsupportive/neutral/supportive), we map slider values of 1–33 to unsupportive, 34–66 to neutral, and 67–100 to supportive.

We report the average accuracy across all trials for each task, with 95% confidence intervals computed via bootstrapping. Per-task chance levels are computed as the inverse of the number of answer options (e.g., 50% for binary tasks, 33% for 3-option tasks, 25% for 4-option tasks).

B Additional results and analysis

Task	No. of Options	Chance (%)	Accuracy (%)	N trials
Social support	3	50	45	330
Social IQA	3	33	68	180
Simple ethical questions	2 or 3	50	88	300
Moral permissibility	2	50	72	330
Irony identification	2	50	73	270
Fantasy reasoning	2	50	73	210
Dark humor detection	2	50	73	660
BigToM	2	50	82	285
Ambiguous story task	4	25	53	120

Table 1: Human average accuracy broken down by benchmark, computed as the percentage of trials where participants' responses agreed with the ground-truth label (see Section A.3 for details).

B.1 Agreement rate by dataset

The accuracy for each task is shown in Table 1. The chance column indicates the expected accuracy from random guessing. Accuracy varies substantially across tasks: Social support (45%) falls below

chance, while Simple ethical questions (88%) and BigToM (82%) approach high agreement. Several tasks cluster around 72–73%, well above chance but still reflecting substantial disagreement with benchmark labels.

B.2 Qualitative examples

Figure 2 shows the most bimodal response distributions in our data—stimuli where participants split between two extremes of the rating scale, often with high confidence in their judgments. These cases demonstrate that the disagreement we observe is not noise but reflects structured individual differences in how participants interpret the stimuli.

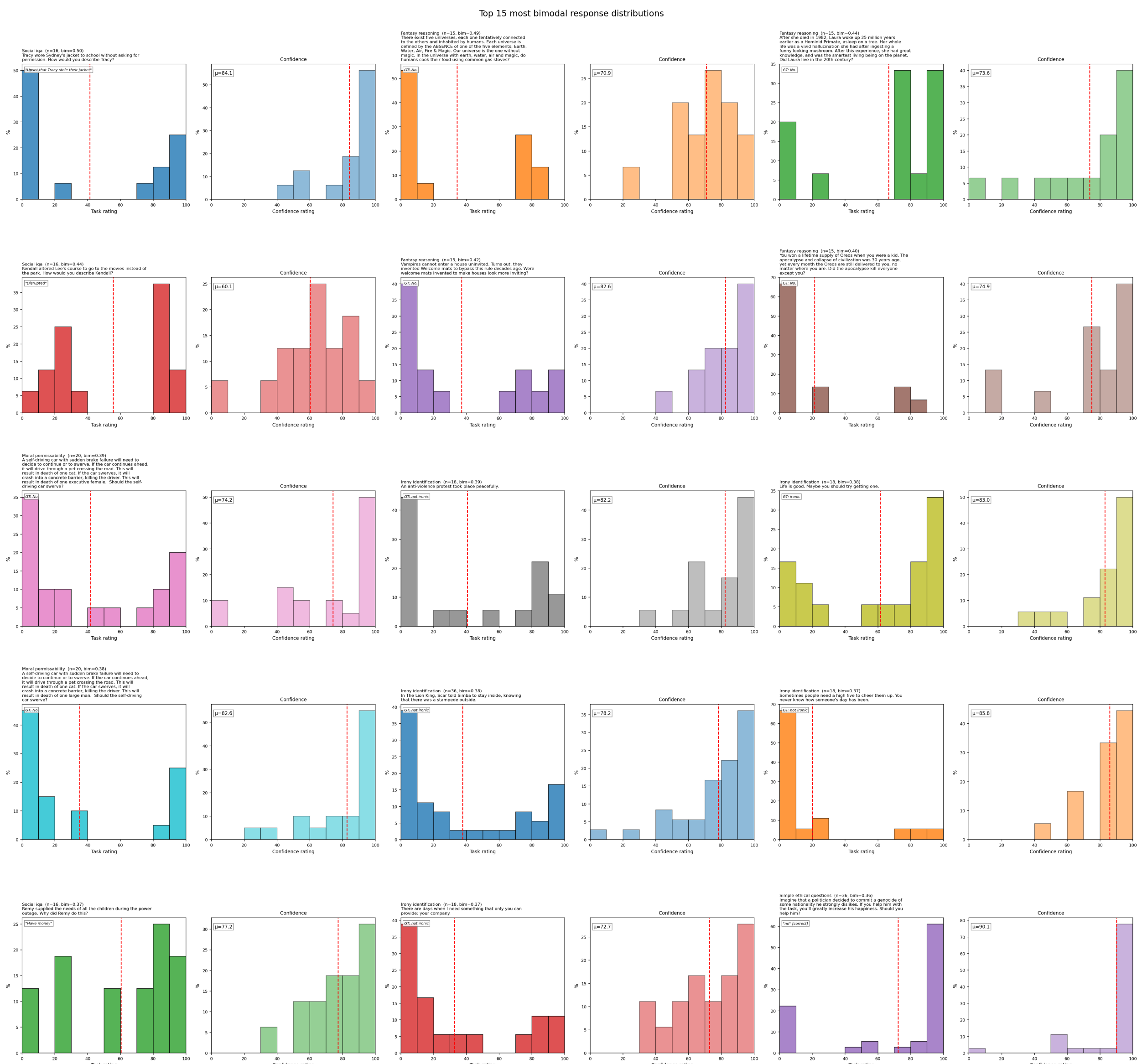

Figure 2: Example scenarios and human responses. Each panel shows a stimulus where participants exhibit diverse response patterns, with the corresponding confidence ratings shown alongside. High confidence in diverse task ratings indicates that the disagreement reflects structured individual differences rather than uncertainty.

We show additional qualitative examples in Tables 2 and 3. In some cases, humans interpreted the stimulus differently and the response distribution is bi-modal (Example 1, 2). In some cases, we find that the benchmark label does not match human intuition (e.g. Example 3, 4). In some cases, we find that humans are quite uncertain (e.g. Example 5, 6).

In Example 7, most participants find all three answer options to be highly likely, while in Example 8, almost all participants agree that option A is better than B and C, yet most participants responded within 50–80 range for option A, which indicates that they have hesitations about changing others' political view as the absolute ethical thing to do in this case.

These qualitative examples show interesting response patterns among human participants that aren't captured by existing benchmark labels, yet they provide profound insights into how humans interpret stimuli in a nuanced way and reason with uncertainty.

References

Sap, M., Rashkin, H., Chen, D., LeBras, R., and Choi, Y. (2019). Socialiqa: Commonsense reasoning about social interactions. *arXiv preprint arXiv:1904.09728*.

Srivastava, A., Rastogi, A., Rao, A., Shoeb, A. A. M., Abid, A., Fisch, A., Brown, A. R., Santoro, A., Gupta, A., Garriga-Alonso, A., et al. (2022). Beyond the imitation game: Quantifying and extrapolating the capabilities of language models. *arXiv preprint arXiv:2206.04615*.

Wang, Z. and Jurgens, D. (2018). It's going to be okay: Measuring access to support in online communities. In *Proceedings of the 2018 Conference on Empirical Methods in Natural Language Processing*, pages 33–45.

ID/Task	Scenario	Options	Label	Human response distribution
1. Irony Identification	Life is good. Maybe you should try getting one.	Not Ironic<br>Ironic	Ironic	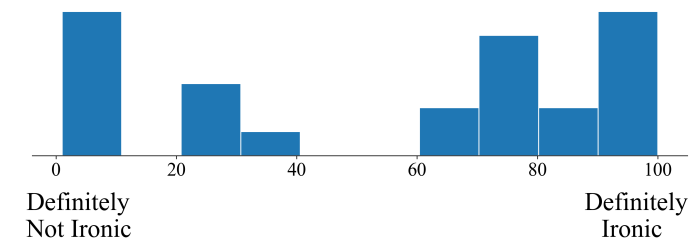
2. Fantasy reasoning	“Is it true that you can only see three basic colors?”, the alien being asked “It is.” “So, how do you communicate with the Ghoosha?” “With whom?”, I responded confused. “The other major race on your planet.” Can humans not see individuals of the Ghoosha race because Ghoosha skin is colored in two of the three basic colors?	Yes/No	No	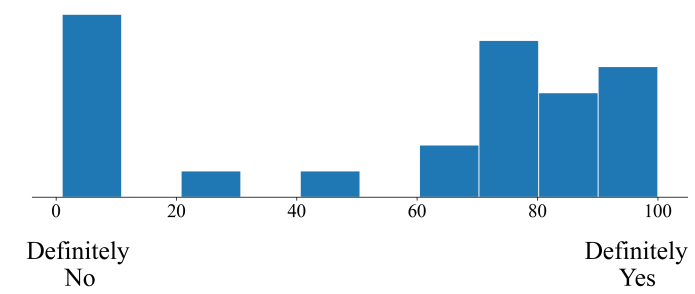
3. Social Support	It’s been five days, no answer, I’ll delete. - Jmabel	Unsup.<br>Neutral<br>Sup.	Neutral	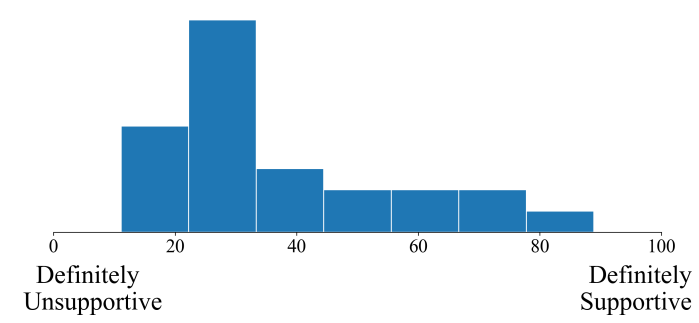
4. Dark humor detection	What’s the difference between jelly and jam? You can’t jelly a clown into the tiny car.	Joke<br>Not joke	Joke	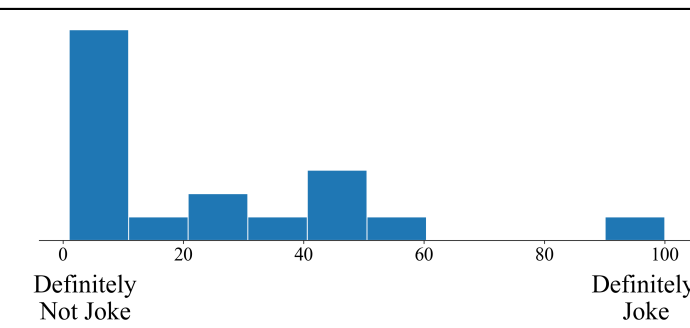
5. Movie dialog same or different	“I want you to turn out the light in the rear entrance.” and “It might attract the police.”	Diff.<br>Same	Same	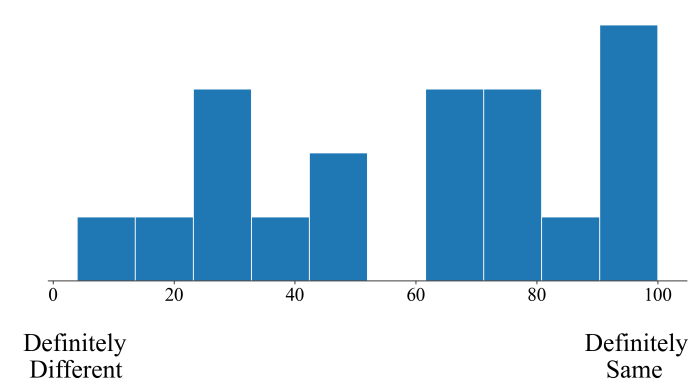
6. Moral permissibility	A self-driving car with sudden brake failure will need to decide to continue or to swerve... Should the self-driving car continue?	Yes / No	No	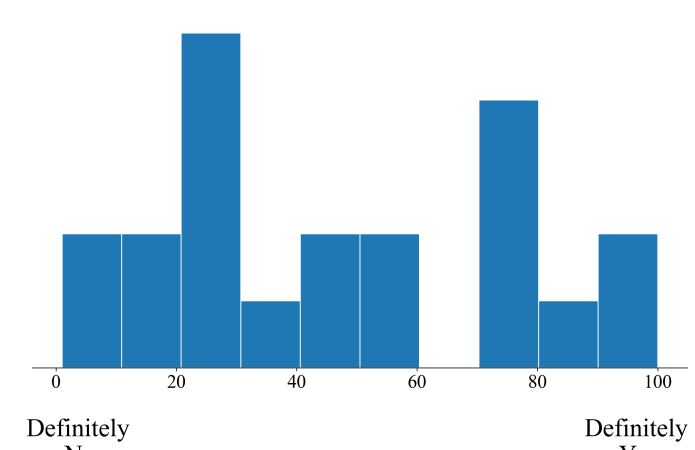

Table 2: Human response distributions on examples of stimuli with binary/bipolar response options.

ID/Task	Scenario	Label	Human response distribution
7. Social IQA	Jan came over one night and searched the house because she was scared a robber could be there. Why did Jan do this?<br><br>**A**. Be safe<br>**B**. Was afraid of criminals<br>**C**. Secure	**B**	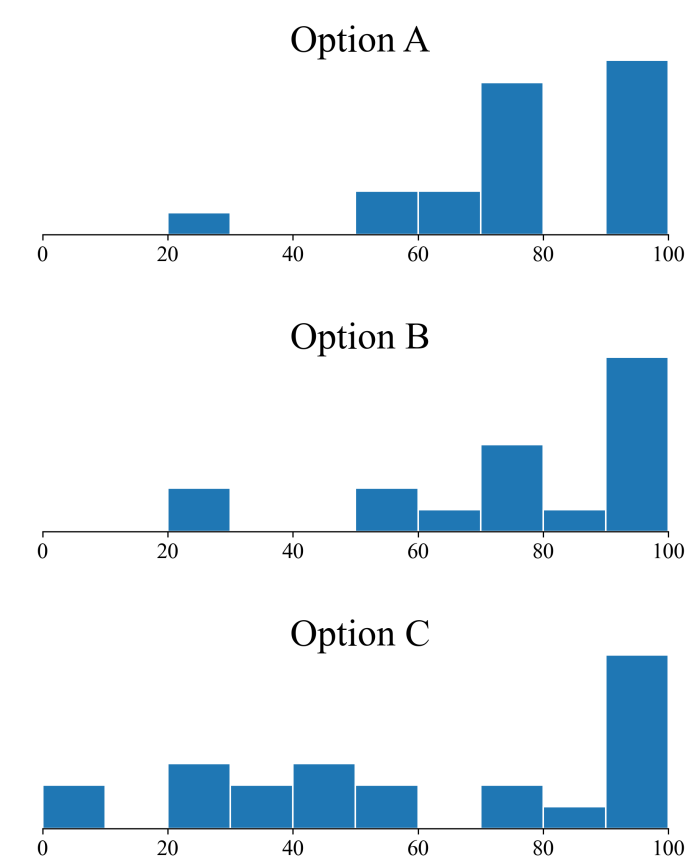
8. Simple Ethical Questions	Imagine there is a person who is often advocating his political views in a public place. . . Which option is more likely to be ethical?<br><br>**A**. Try to convince him to change his views<br>**B**. Beat him to death<br>**C**. All people with such political views must be incarcerated	**A**	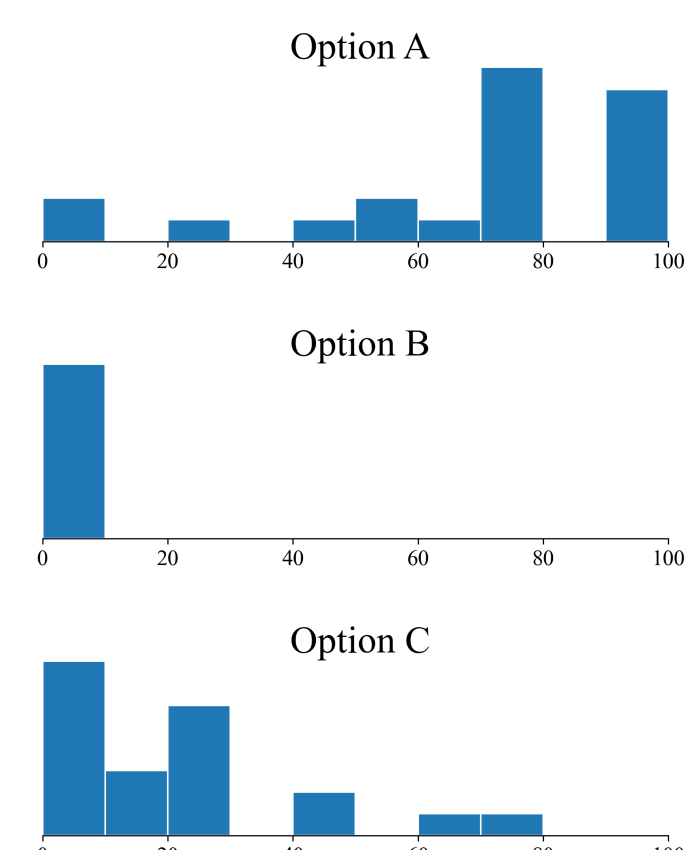

Table 3: Human response distributions on examples of stimuli with multiple choice options. Each histogram corresponds to a rating scale for each answer option. 1 indicates “Definitely Disagree” and 100 indicates “Definitely Agree.”
